# Optimizing Statin Therapy in Older Adults: A Systematic Review of Dosing, Titration, and Combination Strategies

**DOI:** 10.1007/s11357-025-01772-w

**Published:** 2025-07-16

**Authors:** Anna Artner, Romána Zelkó, Balázs Hankó

**Affiliations:** 1 Center of Pharmacology and Drug Research & Development, Semmelweis University, Budapest, 1085 Hungary; 2 University Pharmacy Department of Pharmacy Administration, Semmelweis University, Budapest, 1085 Hungary

**Keywords:** Statin therapy, Dose adjustment, Titration, Elderly, Combination therapy, Cardiovascular disease

## Abstract

Statins are crucial in lipid-lowering therapy for reducing atherosclerotic cardiovascular disease (ASCVD) risk. However, rhabdomyolysis, a rare but severe side effect, disproportionately affects frail, older adults with polypharmacy and multiple comorbidities. Current guidelines for adjusting statin dosages in older adults lack clarity. To evaluate statin use in the geriatric population, assess benefit-risk ratios, and analyze dosage regimens through a systematic literature review. A comprehensive search was conducted in PubMed, Scopus, Web of Science, and EBSCO databases. Eleven original studies involving 566,509 patients were included. The review was registered on PROSPERO (CRD42024587259). Statin therapy was associated with a lower risk of cardiovascular disease compared to placebo. When adjusted during treatment, higher-intensity statins appeared to provide greater benefits than fixed low-dose statins, while the likelihood of side effects remained similar. Using ezetimibe and statins further improved outcomes and was linked to fewer side effects. Adherence-improving techniques were crucial, especially in older adults. Individualized statin dosing, considering titration, combination therapy, and adherence strategies, is essential for maximizing benefits and minimizing risks in older adults. While statins effectively reduce CVD risk, careful consideration of patient-specific factors is necessary when prescribing to older adults.

**Graphical Abstract:**

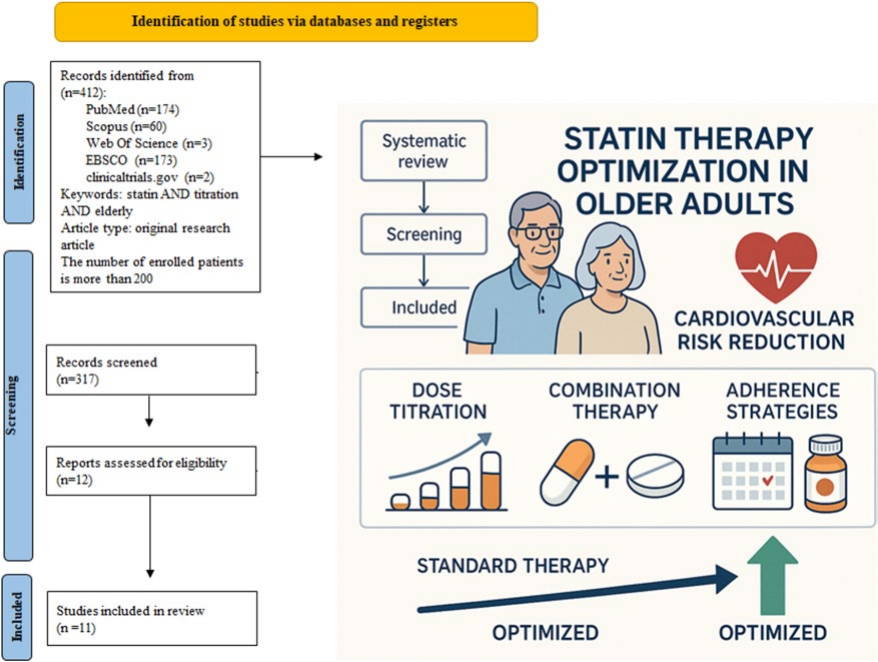

## Introduction

Cardiovascular disease (CVD) is a significant global health concern, contributing substantially to mortality and morbidity worldwide. Managing modifiable risk factors for CVD is crucial, with dyslipidemia control playing a central role in both primary and secondary prevention strategies. The use of lipid-lowering therapies, particularly statins, has proven highly effective in reducing the risk of atherosclerotic cardiovascular disease (ASCVD) [1]. As individuals age, the incidence of cardiovascular events increases, with men typically affected after 65 years and women after 75 years. Older adults present a distinct patient population characterized by age-related physiological changes, multiple comorbidities, complex medication regimens, and often cognitive impairment [2].

Optimal selection of therapy is crucial for this patient demographic due to these factors. Certain guidelines utilize diverse risk assessment tools to categorize patients accurately into the appropriate therapeutic cohort in alignment with the recommendations. The most recent recommendations from the European Society of Cardiology (ESC) on managing lipid values suggest that in managing ASCVD in older individuals, it is crucial to administer statin therapy in a manner consistent with younger patients. Additionally, for primary prevention in individuals aged over 75 years, initiating statin treatment may be warranted, especially for those at high risk or above. When faced with significant renal impairment or potential drug interactions, it is prudent to commence statin therapy at a low dose and incrementally adjust it to achieve the desired low-density lipoprotein cholesterol (LDL-C) treatment goals [3]. However, the American Heart Association’s (AHA) recommendations for primary prevention for adults older than 75 years old suggest that the initiation of moderate-intensity statin therapy for individuals with LDL-C levels ranging from 1.81 to 4.89 mM can be a reasonable approach. It is also crucial to adopt a patient-centered perspective and carefully evaluate factors such as functional decline, multimorbidity, frailty, and life expectancy. If these considerations suggest that the risks outweigh the potential benefits of statin therapy, discontinuation should be contemplated [4]. The ESC recommendations on secondary prevention already emphasize the use of combination therapy, especially for patients in very high-risk groups. In secondary prevention, the AHA highlights the importance of carefully evaluating the potential benefits in relation to the risks and adverse effects before initiating statin therapy in patients aged 75 or older. This approach ensures thorough consideration of the individual’s health status and risk factors to optimize treatment decisions and outcomes [3]. Table 1 compares and summarizes the main points of the European and American guidelines. While previous reviews have predominantly focused on the general efficacy and safety of statin therapy in the population of older adults, this review emphasizes the critical importance of titration and individualized dosing strategies [5–15]. This systematic literature review aims to assess the benefit-risk ratio of statin use specifically in older adults, highlighting the need for tailored dosing based on individual patient profiles.

**Table 1:** International guidelines on statin therapy in geriatric patients [3, 4]

	**ESC/EAS**	**AHA/ACC**
Age criteria for older adults	> 65 years	> 75 years
Risk assessment tools	• SCORE • CAC • Lipid analyses for CVD risk estimation	• SCORE • CAC • Clinician-patient risk discussion
Statin therapy recommendations (primary and secondary prevention)	**Primary prevention:** • Older individuals with ASCVD should receive statin treatment similarly to younger patients • Considering starting statin treatment for primary prevention in individuals over 75 years old may be appropriate if they are at high risk or above • If there is notable renal impairment or a risk of drug interactions, it is advisable to begin statin treatment at a low dose and gradually increase it to reach LDL-C treatment targets **Secondary prevention:** • In cases where patients at very high risk fail to reach their target despite maximum tolerated doses of statin and ezetimibe, combining with a PCSK9 inhibitor is recommended for secondary prevention	**Primary prevention:** • For adults over 75 with LDL-C levels of 70–189 mg/dL (1.81–4.89 mM), starting moderate-intensity statins may be reasonable • Consider discontinuing statins if functional decline, multimorbidity, frailty, or reduced life expectancy outweigh potential benefits • For adults aged 76–80 with LDL-C levels of 70–189 mg/dL (1.81–4.89 mM), measuring CAC to identify those with a score of zero might justify avoiding statins **Secondary prevention:** • In patients over 75 with clinical ASCVD, consider starting moderate- or high-intensity statin therapy after assessing risk reduction potential, adverse effects, drug interactions, frailty, and patient preferences • If tolerating high-intensity statins, continue after similar evaluation • To summarize, before starting statin therapy in patients over 75 with ASCVD, weigh the potential benefits against the risks and adverse effects • For adults over 75 with diabetes, continuing statin therapy is reasonable • Starting statins may also be reasonable after discussing benefits and risks • They are at high risk and likely benefit from statins, despite potential drawbacks due to age and increased adverse events
Recommendation for monitoring	• Assessing therapy response typically occurs 6–8 weeks after starting treatment • Subsequent follow-up intervals are often set at 6–12 months, but they are somewhat arbitrary • At a minimum, LDL-C levels should be checked when available, although a complete lipid profile including HDL-C and TGs may lead to better treatment decisions • Additionally, non-HDL-C or ApoB should be considered as secondary treatment targets • Regular lipid monitoring not only aids in treatment decisions but also supports patient adherence to lifestyle changes or medications, as evidenced by various studies	• Assess adherence to lifestyle changes and the effects of LDL-C-lowering medication by measuring fasting lipids and safety indicators 4 to 12 weeks after starting or adjusting statin doses, and then every 3 to 12 months as necessary for monitoring adherence and safety

*ESC/EAS *European Society of Cardiology/European Atherosclerosis Society, *AHA/ACC *American Heart Association/American College of Cardiology, *SCORE *Systematic Coronary Risk Evaluation, *CAC *Coronary Artery Calcium, *CVD *Cardiovascular Disease,* ASCVD* Atherosclerotic Cardiovascular Disease, *LDLC* Low-Density Lipoprotein Cholesterol, *PCSK9* Proprotein Convertase Subtilisin/Kexin type 9, *HDL *High-Density Lipoprotein Cholesterol, *TG *Triglycerides, non-*HDL*-C Non High-Density Lipoprotein Cholesterol, ApoB Apolipoprotein B

## Methods

### Search strategy and eligibility criteria

**Fig. 1:**
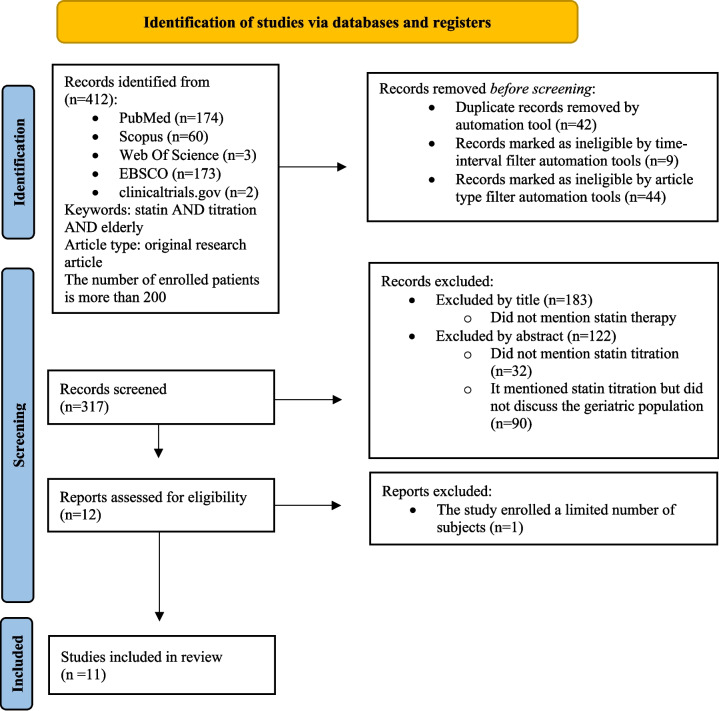
PRISMA 2020 flow diagram

This systematic review was conducted following the Preferred Reporting Items for Systematic Reviews and Meta-Analyses (PRISMA) guidelines, ensuring adherence to the PRISMA 27-item checklist [16]. Figure 1 visually represents the identification of studies via the PRISMA flow diagram. We performed a comprehensive literature search across multiple databases, including PubMed, Scopus, Web of Science, and EBSCO, to identify original research articles focusing on statin therapy in the population of older adults. The search strategy employed specific keywords such as “statin,” “titration,” and “elderly,” and the search was limited to studies published from 1994 to January 2024.

Inclusion criteria were (1) original research articles written in English addressing statin therapy in patients aged ≥ 65 years; (2) studies examining the impact of statin dosing regimens, including titration and combination therapies; and (3) studies reporting clinical outcomes or benefit-risk assessments. Exclusion criteria included non-original research articles (e.g., reviews and case reports), studies with insufficient data, and those with fewer than 100 participants.

The study selection process was conducted systematically by two independent reviewers (A.A. and R.Z.). The initial screening phase involved evaluating titles and abstracts, followed by a comprehensive full-text review of studies meeting the eligibility criteria. In cases of discrepancies between the two primary reviewers, a thorough discussion was initiated to reach a consensus. When agreement could not be achieved through discussion alone, a third reviewer (B.H.) was consulted to provide additional expertise and facilitate resolution of the disagreement. The final inclusion decision was based on a majority consensus among the three reviewers. It is noteworthy that only one instance during the selection process necessitated the involvement of the third reviewer for consensus.

### Data extraction

Data extraction was performed independently by two reviewers (A.A. and R.Z.) using a standardized data extraction form. The following information was collected from each selected study: study design, population characteristics, statin intervention details, dosage regimens, number of participants, duration of follow-up, reported outcomes, and any adverse events associated with statin therapy. All extracted data were compiled into a summary table to facilitate comparison and synthesis of the findings. Disagreements were resolved through consensus or consultation with the third reviewer (B.H.).

### Protocol and registration

This review was conducted in accordance with the guidelines for systematic reviews and meta-analyses. The protocol for this review was registered with PROSPERO, the international prospective register of systematic reviews, under the registration number (registration number.

CRD42024587259) (Centre for Reviews and Dissemination, University of York;

http://www.crd.york. ac.uk/PROSPERO) prior to commencing the study.

### Quality assessment within studies and overall quality assessment

The methodological quality of the included studies was evaluated using the National Institutes of Health Quality Assessment Toolkits for Observational Cohort and Cross-Sectional Studies [17]. This toolkit comprises 14 questions that assess various elements related to bias risk, transparency, and confounding factors. These elements include the study question, population characteristics, participation rate, inclusion criteria, sample size, timing of exposure relative to the outcome, adequate time frame, different exposure levels, exposure measures, outcome measures, blinding of outcome, and loss to follow-up. Not all of the selected studies could be evaluated by the NIH toolkit. The reasons for exclusion included a lack of necessary data, non-observational study designs, short study duration or limited follow-up, and a focus on non-clinical outcomes. Any disagreements about the methodological quality of the included studies were resolved through discussion until consensus was achieved. Table 2 shows the results of methodological quality assessment within studies.

**Table 2:** Methodological quality assessment within studies, evaluated using the NIH toolkit

	Study Question	Study population	Participation Rat	Inclusion Criteria	Sample Size	Exposure Prior to Outcome	Sufficient Time Frame	Different Levels of Exposure	Exposure Measures	Multiple Exposures	Outcome Measures	Blinding of Outcome	Loss to Follow-Up	Confounding
Lipka et al. [5]	✓	✓	✓	✓	✓	✓	✓	✓	✓	✓	✓	✓	x	✓
Neil et al. [6]	✓	✓	✓	✓	✓	✓	✓	✓	✓	x	✓	✓	x	✓
Koren et al.[7]	✓	✓	✓	✓	✓	✓	✓	✓	✓	✓	✓	✓	x	✓
Zieve et al. [8]	✓	✓	✓	✓	✓	✓	✓	✓	✓	✓	✓	✓	x	✓
Campitelli et al. [12]	✓	✓	✓	✓	✓	✓	✓	✓	✓	✓	✓	x	x	✓

✓: element reported appropriately in the study, x, element not mentioned in the study

To evaluate the overall quality of evidence in the present systematic review, a modified version of the Grading of Recommendations Assessment, Development, and Evaluation (GRADE) system was used [18]. The review considered several factors to assess the quality of evidence for lipid and clinical outcomes in studies on statin therapy in older adults. These factors included the strength of evidence for lipid and clinical outcomes in each selected study, study design, directness, and precision. The evidence was then rated as low, moderate, or high. Table 3. shows the results of the modified version of the GRADE system.

**Table 3:**
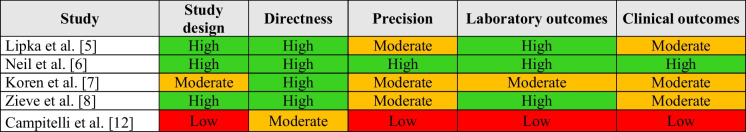
GRADE Assessment of Evidence Quality for Laboratory and Clinical Outcomes in Studies on Statin Therapy in Older Adults

**Study Design**: Indicates the overall strength of the study design (e.g., High for RCTs, Moderate for cohort studies, Low for observational studies); **Directness**: Evaluates whether the study directly answers the research question without too many indirect assumptions;** Precision**: Indicates the precision of the study's results

### Data synthesis

Due to the heterogeneity of study designs and outcomes, a narrative synthesis approach was adopted. Studies were grouped by intervention type (e.g., statin monotherapy and combination therapy) and key outcomes (e.g., lipid profile changes and cardiovascular events). Where possible, quantitative data were presented in summary tables to facilitate comparison across studies.

## Results

The results of studies included in this review using PRISMA are summarized in Table 4 [16]. The selected studies were analyzed based on various criteria including study design, aim, statin intervention, statin dose, number of patients, study duration, comorbidities, gender, study results, and clinical outcome. As shown in Table 4, laboratory outcomes were prominently highlighted across the studies, although not all studies included clinical outcomes. Dementia and cognitive impairment as comorbidities did not serve as exclusion criteria for study inclusion. Due to the heterogeneity of study designs and populations, a meta-analysis was not feasible. However, we have conducted a narrative synthesis to highlight key trends and relationships across studies.

**Table 4:** Overview of clinical trials examined in the review

Ref	Study design	Aim	Intervention	Dose of statin	No. of P. (men and women) (*n* =)	Time of study	Comorbidities	Results	Clinical outcome
**Lipka et al. **[5]	Multicenter, randomized, double-blind, placebo-controlled, balanced parallel-group trial	To compare the efficacy and safety of statin monotherapy in combination with ezetimibe in older and younger adults with primary hypercholesterolemia	Statin alone or ezetimibe (10 mg) + statin	Lovastatin or pravastatin (10, 20, or 40 mg), simvastatin, or atorvastatin (10, 20, 40, or 80 mg)	652	12 weeks	Primary hypercholesterolemia	The combination of statin plus ezetimibe showed a greater reduction in LDL cholesterol and TG levels compared to statin monotherapy	There was no recorded clinical outcome
**Neil et al. **[6]	Randomized placebo-controlled trial	To compare the efficacy and safety of atorvastatin in older and younger adults with type 2 diabetes	Atorvastatin or placebo	10 mg/day	1129	3.9 years	Type 2 diabetes, presence of at least one cardiovascular disease risk factor	In the older group, LDL cholesterol levels decreased by 41% with 10 mg of atorvastatin compared to placebo. Modest enhancements were observed in HDL cholesterol and triglyceride levels	Atorvastatin reduced the relative risk of initial major cardiovascular events by 38% in older adults. Over the course of the study, 89 older patients and 54 younger patients died
**Koren et al. **[7]	Population-based, randomized, open-label, comparative study	Dispelling doubts about the efficacy and safety of high-dose statin therapies in older adults through the ALLIANCE study	Atorvastatin (aggressive treatment group), other lipid-lowering therapy drug (usual care group)	Dose titrated in the aggressive care group to maximum 80 mg/day	1001	53.9 months	Coronary heart disease	By the end of the study, more older adults in the high-dose atorvastatin group met National Cholesterol Education Program—Adult Treatment Panel (NCEP-ATP) III cholesterol targets compared to those receiving standard care	Older adults undergoing a structured atorvastatin regimen witnessed a 27% decrease in the primary endpoint risk, along with a 52% decrease in the risk of cardiac death and nonfatal MI
**Zieve et al. **[8]	Multicenter, randomized, double-blind, parallel-arm study	Assess the efficacy and tolerability of adding ezetimibe to atorvastatin versus increasing atorvastatin dosage alone in older adults (aged 65 years and older) with hypercholesterolemia at high risk for coronary heart disease	Atorvastatin alone or atorvastatin + ezetimibe	Atorvastatin alone (10 mg daily, up-titrated to 40 mg/day), atorvastatin + ezetimibe (atorvastatin 10 mg/day)	1053	12 weeks	Hypercholesterolemia, a part of the study population has atherosclerotic vascular disease	Atorvastatin 10 mg + ezetimibe group showed superior LDL reduction, increased HDL, and decreased triglycerides compared to titrated atorvastatin. Individuals in this group also reached LDL targets faster at weeks 6 and 12 than the atorvastatin-only group	There was no recorded clinical outcome
**Bays et al. **[9]	Post hoc analysis of two multicenter, 6-week, double-blind, randomized, parallel-group trials	Investigate how age, gender, and race influence the effectiveness of adding ezetimibe to atorvastatin compared to up-titrating atorvastatin alone in patients at moderate to high risk of coronary heart disease	Atorvastatin alone or atorvastatin + ezetimibe	High CHD risk subjects: atorvastatin 40 mg plus ezetimibe 10 mg daily, or up-titrated to atorvastatin 80 mg/day. Moderately high CHD risk subjects: atorvastatin 20 mg plus ezetimibe 10 mg daily, or atorvastatin 40 mg/day	775 (≥ 65 years old patients: 268)	6 weeks	Coronary heart disease, diabetes, metabolic syndrome	For all subgroups, ezetimibe + atorvastatin provided greater percentage reductions in LDL-C, total cholesterol, triglycerides, non-HDL cholesterol, and apolipoprotein B compared to increasing atorvastatin dosage alone	There was no recorded clinical outcome
**Stender et al. **[10]	Open-label, multicenter extension study	To evaluate the long-term efficacy, safety, and tolerability of pitavastatin in older adults with primary hypercholesterolemia or combined (mixed) dyslipidemia	Pitavastatin	2 mg/day, up-titrated to 4 mg/day	545	60 weeks	Primary hypercholesterolemia or combined (mixed) dyslipidemia	Investigate how age, gender, and race influence the effectiveness of adding ezetimibe to atorvastatin compared to up-titrating atorvastatin alone in patients at moderate to high risk of coronary heart disease	There was no recorded clinical outcome
**Constance et al. **[11]	Post hoc analysis of the 12-week, multicenter, randomized, double-blind, parallel-arm, ZETELD study	To compare the efficacy of atorvastatin 10 mg plus ezetimibe versus titration to atorvastatin 40 mg in achieving European and Canadian guideline lipid targets in high-risk subjects aged 65 years or older	Atorvastatin alone or atorvastatin + ezetimibe	Atorvastatin alone (10 mg daily, up-titrated to 40 mg/day), atorvastatin + ezetimibe (atorvastatin 10 mg/day)	1053	12 weeks	Hypercholesterolemia, a part of the study population has atherosclerotic vascular disease	Atorvastatin + ezetimibe achieved significantly greater LDL-C, non-HDL-C, and Apo B targets compared to atorvastatin 40 mg titration. Canadian targets were met more often with combination therapy versus atorvastatin 20 mg (6 weeks) or 40 mg (12 weeks). In high-risk patients, both groups had similar odds of meeting most targets at 6 and 12 weeks	There was no recorded clinical outcome
**Campitelli et al. **[12]	Retrospective cohort study	To explore the impact of statin dosage on one-year survival and hospital admissions due to cardiovascular events among individuals residing in long-term care facilities	Atorvastatin, rosuvastatin, simvastatin	Intensive dose: atorvastatin (40 mg or more daily). Rosuvastatin (20 mg or more daily), simvastatin (80 mg or more daily)	21,808	1 year	Atherosclerotic cardiovascular disease	In this population-based cohort, residents of long-term care facilities who were prescribed intensive-dose statins showed no significant difference in 1-year outcomes compared to those prescribed moderate-dose statins	After 1 year, moderate-dose prescriptions were associated with 26.4% deaths and 11.4% cardiovascular hospital admissions, while intensive-dose prescriptions had 25.6% deaths and 11.4% hospital admissions
**Musich et al. **[13]	Retrospective cohort study	The study aimed to evaluate statin usage across therapy intensities in males and females and to analyze how varying statin intensities affected overall survival rates in both genders	Statin, non-statin	High intensity, moderate intensity, low intensity, nonadherent	94,240 (49,530 men and 44,710 women)	2 years	Cardiovascular disease + CCI conditions and EBM conditions	In both men and women, mortality rates rose as statin intensity decreased. For men, any statin use was better than none, while women benefited significantly from moderate to high-intensity statins in reducing mortality	High-intensity statins correlated with more CVD hospitalizations in men and women, while lower statin use had fewer hospitalizations. Non-adherence had the highest proportion of CVD hospitalizations
**Mack et al. **[14]	Cross-sectional study	To assess how common statin use is among long-stay nursing home residents with life-limiting illnesses and to identify factors that may be linked to the use of statin medications in this population	Atorvastatin, rosuvastatin, simvastatin, fluvastatin, lovastatin, pitavastatin, pravastatin	High intensity statin therapy: atorvastatin (40 or 80 mg daily), rosuvastatin (20 or 40 mg daily), simvastatin (80 mg daily) Low-moderate statin therapy: atorvastatin (10 or 20 mg daily), fluvastatin (20 or 40 mg daily), lovastatin (10, 20, 40, or 80 mg daily), pitavastatin (1, 2, or 4 mg daily), pravastatin (10, 20, 40, or 80 mg daily), rosuvastatin (5 or 10 mg daily), simvastatin (5, 10, 20, or 40 mg daily)	424,212	90 days	Serious illness, in palliative care, life expectancy is lower than 6 months	34% of residents with life-limiting illnesses received statin prescriptions. For ages 65–75, the rate was 44% (11.1% high-intensity), while for over 75 s, it was 31.1% (5.4% high-intensity)	Factors negatively associated with statin prescriptions in 65–75 year olds were severe cognitive/functional impairment, liver disease, and limited prognosis indicators. For over 75 years, old residents and negative associations included female gender, nursing home residency > 1 year, severe impairment, serious illnesses, and limited prognosis
**Spencer-Bonilla et al. **[15]	Retrospective longitudinal study	To investigate the patterns and factors influencing the utilization of statin therapy among older adults diagnosed with stable atherosclerotic cardiovascular disease	Atorvastatin, rosuvastatin, lovastatin, simvastatin, fluvastatin, pitavastatin, pravastatin	I: atorvastatin (40–80 mg), rosuvastatin (20–40 mg), lovastatin (80 mg), simvastatin (80 mg) II: atorvastatin (10–40 mg), fluvastatin (80 mg), lovastatin (40–60 mg), pitavastatin (2–4 mg), pravastatin (40–80 mg), rosuvastatin (5–19 mg), simvastatin (20–40 mg) III: atorvastatin (< 10 mg), rosuvastatin (< 5 mg), fluvastatin (< 80 mg), lovastatin, pravastatin (< 40 mg), pitavastatin (1 mg), simvastatin (< 20 mg)	24,651	11 years	Atherosclerotic cardiovascular disease + hypertension, diabetes, heart failure, renal disease, and dementia	Despite an increase in prescriptions for moderate/high intensity statins among adults over 75 years old over time, less than half of the patients (45%) received such therapy in 2018. Women, individuals with heart failure, those diagnosed with dementia, and patients classified as underweight were less likely to be prescribed moderate/high intensity statins	There was no recorded clinical outcome

### Efficacy of statin therapy in older adults

The comprehensive results from various studies shed light on the efficacy and outcomes of different lipid-lowering therapies with statins, particularly in the population of older adults. Clinical trials and studies on the safe use of statins and dose titration began in the early 2000s. The 2002 American College of Cardiology, American Heart Association, and National Heart, Lung and Blood Institute (ACC/AHA/NHLBI) Clinical Advisory on the Use and Safety of Statins highlights the safety and efficacy of statins in the vast majority of patients who receive them [19].

The findings indicate that statins are generally effective in reducing cardiovascular events across various subgroups of older adults. For example, in a randomized placebo-controlled trial, the efficacy and safety of atorvastatin were compared in older and younger adults with type 2 diabetes. At study end, atorvastatin 10 mg decreased LDL cholesterol by 41% in the older group versus placebo, with modest improvements in High-density lipoprotein (HDL) cholesterol and triglycerides [6]. A 1% increase in HDL cholesterol levels can decrease cardiovascular risk by as much as 2% [20]. In the group aged 65 years and older, adverse events related to treatment were reported in 25% of patients receiving atorvastatin compared to 24% of those on placebo. Serious adverse events occurred in 1.2% of atorvastatin-treated patients and 1.6% of placebo-treated patients, with myalgia reported in 3.5% of atorvastatin patients and 4.8% of placebo patients. Notably, no cases of rhabdomyolysis were documented during the study [6].

The efficacy of statin treatment in older adults is a multifaceted issue that can be assessed not only through laboratory measures, such as reductions in LDL cholesterol, but also by examining clinical outcomes, including the prevention of cardiovascular events. This dual approach is crucial because the full benefits of statin therapy, particularly in reducing major cardiovascular incidents, may not become evident until approximately 2.5 years of continuous use. However, early indicators, such as improved laboratory parameters, can often be observed as soon as 12 weeks into the treatment [21]. Considering the advanced age of the patient populations in the included studies, an extended follow-up period is necessary to comprehensively assess the long-term safety profile of statin therapy. The variability in follow-up durations across the included studies may introduce potential bias in the overall safety assessment. Studies with insufficient follow-up periods should be interpreted with caution, as they may not capture delayed adverse effects or long-term outcomes associated with statin use in the population of older adults.

The use of atorvastatin in individuals aged 65 years and older has demonstrated significant efficacy in reducing the risk of major cardiovascular events. In a study involving 1000 older adults, atorvastatin was shown to prevent 48 initial major cardiovascular events over a 4-year period, with a Number Needed to Treat (NNT) of 21 to prevent one primary event [6].

The premature termination of the trial 2 years earlier than planned resulted in reduced statistical power, potentially compromising the robustness of the findings. While the study reported a 49% reduction in stroke incidence, this result only achieved borderline statistical significance. The observed trend suggests a potential protective effect against stroke, but the lack of formal statistical significance necessitates cautious interpretation of these results.

It is important to note that the study’s findings have limited generalizability to the very elderly population, as individuals aged 75 and older were excluded from the trial at randomization. The early cessation of the study may have influenced the observed outcomes, and a full-duration trial could have yielded different results, either reinforcing or attenuating the reported effects [6].

Given the limitations of the truncated study period and the resulting incomplete data set, the findings provide a promising but preliminary indication of atorvastatin’s potential to reduce cardiovascular events in older diabetic patients. Further research with larger sample sizes and longer durations would be necessary to confirm these results and establish their clinical significance.

The ALLIANCE study, which focused on aggressive atorvastatin treatment in older adults, demonstrated a 27% reduction in the relative risk of primary cardiovascular events. This was accompanied by a significant 52% decrease in the composite endpoint of cardiac death and nonfatal myocardial infarction (MI), driven primarily by a 57% decline in nonfatal MI risk. Additionally, there was a notable 3333% relative risk reduction in cardiac revascularization for patients aged 65–75, underscoring the effectiveness of aggressive statin therapy in older adults.

Participants under 65 years of age demonstrated significant cardiovascular benefits, with a 37% relative risk reduction in the combined endpoint of cardiac mortality and nonfatal myocardial infarction (MI) (*p* = 0.008). However, patients aged 65–75 years exhibited even more pronounced risk reductions for the primary composite outcome, nonfatal MI, and the combined endpoint of cardiac mortality and nonfatal MI. These results indicate that the titrated atorvastatin regimen provided at least equivalent, and potentially superior, cardiovascular protection for older patients compared to their younger counterparts [7].

Further supporting these findings, a retrospective cohort study observed that mortality rates increased as statin intensity decreased, with both males and females benefiting significantly from high- and moderate-intensity statins. The study also highlighted the critical importance of adherence to statin therapy, as individuals who were nonadherent exhibited the highest hospitalization rates. Even minimal statin usage was found to be better than no statin therapy at all, particularly for males. However, high-intensity statins were associated with increased cardiovascular disease hospitalizations, while lower intensity or no statin use corresponded with reduced hospitalization rates in both genders [13].

On the other hand, the use of statins in specific subpopulations, such as those in palliative care with a life expectancy of less than 6 months, presents unique challenges. One study included in the review focused on this population, finding that 34% of residents with life-limiting illnesses were prescribed statins. Among those aged 65–75, 44% received statin therapy (11.1% at high-intensity), while only 31.1% of those over 75 were prescribed statins (5.4% at high-intensity). The study identified several factors negatively associated with statin prescribing in these patients, including severe cognitive or functional impairment, liver disease, and limited prognosis. For residents over 75 years old, factors such as female gender, extended nursing home residency, and severe illnesses further reduced the likelihood of being prescribed statins [14].

The evidence underscores the importance of tailoring statin therapy to individual patient characteristics, particularly in geriatric populations. While statins demonstrate significant efficacy in reducing cardiovascular risk, their prescription in patients with limited life expectancy or multiple comorbidities necessitates a thorough risk–benefit analysis and alignment with comprehensive treatment goals. This approach ensures that statin therapy is optimized for each patient’s unique clinical profile and overall health objectives.

### Importance of dose titration

Given the established safety of statin, the focus in clinical practice can shift toward optimizing dosing regimens, particularly in the population of older adults [22]. Interestingly, despite the widespread use of statins, relatively few studies have specifically examined the effects of dose titration in older adults. This is notable because there is still insufficient evidence to fully support the use of statins for primary prevention in older adults, yet the available studies suggest that dose titration could play a crucial role in reducing the relative risk of initial major cardiovascular events.

For instance, the population-based, randomized, open-label ALLIANCE study demonstrated that aggressive dose titration in older adults could effectively achieve target LDL-C levels with minimal adverse events. At baseline, the average LDL-C levels for the older cohort were 3.77 mM in the atorvastatin group and 3.73 mM in the usual care group. By the end of the study, these levels had significantly decreased to 2.35 mM and 2.77 mM, respectively. Although adverse events were more common in older individuals, the rates of serious adverse events leading to discontinuation were similar between the younger and older cohorts in both treatment arms [7].

Moreover, a 60-week study on pitavastatin treatment showed that mean LDL-C concentrations in the efficacy cohort were approximately 43% lower compared to baseline. This study also reported significant reductions in total cholesterol, triglycerides, and other markers, with 82.0% of participants experiencing treatment-emergent adverse events (TEAEs). However, serious TEAEs occurred in only 10.4% of patients [10].

Throughout the core study and extension phase, all participants followed a diet low in fat and cholesterol, adhering to the EAS guidelines. This dietary intervention may have contributed to the observed improvements in lipid profiles. However, it is important to note that this study did not assess the effects of pitavastatin on morbidity or mortality, which are crucial endpoints for evaluating the drug’s overall clinical efficacy. Furthermore, due to the non-randomized nature of the study design, direct comparisons of efficacy between the two pitavastatin doses are not statistically valid or appropriate.

A retrospective cohort study revealed that adjusted mortality rates increased as statin intensity decreased, with both males and females benefiting significantly from high- and moderate-intensity statins. Notably, individuals who were nonadherent to statin therapy had the highest hospitalization rates, highlighting the critical importance of adherence. Even minimal statin use was found to be better than no statin therapy at all, particularly in males. While high-intensity statins were associated with increased rates of cardiovascular disease hospitalizations, lower rates were observed with lower or no statin use in both genders [13].

### Combination therapy

As detailed in Table 2, several studies investigated the use of statins not only as monotherapy but also in combination with ezetimibe, a drug that lowers LDL-C by reducing the intestinal absorption of cholesterol [23].

Combining ezetimibe with statins has been shown to enhance the effectiveness of LDL-C reduction across all age groups. In a multicenter, randomized, double-blind, placebo-controlled trial, the combination of ezetimibe and statin proved more effective than statin monotherapy in lowering LDL-C, reducing triglycerides, and increasing HDL-C levels. The reduction in triglycerides was particularly notable with the combination therapy, and HDL-C levels increased by 8–11% with ezetimibe plus statin, compared to a 5–6% increase with statin monotherapy. The combined therapy was well tolerated across patient categories, with a safety profile similar to that of statin monotherapy. However, older adults, particularly those aged 75 and older, experienced a slightly higher incidence of adverse events, primarily muscle-related symptoms such as myalgia, muscle weakness, and cramps, leading to a higher rate of discontinuation in this age group [5].

In a 12-week multicenter, randomized, double-blind, parallel-arm study comparing atorvastatin monotherapy to a combination of atorvastatin and ezetimibe, patients with an initial LDL cholesterol level of 3.36 mM experienced a more significant reduction in LDL cholesterol with the combination therapy at both 6 and 12 weeks. The group receiving atorvastatin 10 mg in combination with ezetimibe had significantly higher rates of achieving LDL cholesterol levels of 1.81 mM or lower compared to those taking atorvastatin 20 mg at week 6 and atorvastatin 20/40 mg at week 12. Improvements in various lipid parameters were more pronounced in the combination group, and adverse events were infrequent and similar across the treatment groups. However, there was a slightly higher incidence of elevated liver enzymes in the group receiving higher doses of atorvastatin compared to the combination therapy group. The study’s findings are encouraging, but it is crucial to note its brevity and the small number of subjects aged 75 and above. These factors should be taken into account when interpreting the results [8].

A post hoc analysis of two multicenter, 6-week, double-blind, randomized trials demonstrated that combining ezetimibe 10 mg with atorvastatin 20 mg for patients at moderately high risk of coronary heart disease (CHD), or atorvastatin 40 mg for those at high CHD risk, resulted in superior reductions in LDL cholesterol, total cholesterol, triglycerides, non-HDL cholesterol, and Apo-B compared to simply doubling the atorvastatin dose. Similarly, a post hoc analysis of the 12-week ZETELD study found that the combination of atorvastatin 10 mg plus ezetimibe was more effective in achieving LDL-C, non-HDL-C, and Apo-B targets than atorvastatin titration to 20 mg. At 6 weeks, a higher percentage of patients reached these targets with the combination therapy, and by 12 weeks, those on ezetimibe plus atorvastatin maintained their target levels better than those who were titrated to 40 mg of atorvastatin. This trend was consistent across high-risk subjects without atherosclerotic vascular disease and was particularly significant for very high-risk individuals [11].

The results of selected studies strongly support the use of combination therapy with ezetimibe and statins as an effective strategy for achieving lipid targets, especially in older adults and those at high risk for cardiovascular events. This approach not only enhances lipid-lowering effects but also maintains a manageable safety profile, making it a valuable option in the management of dyslipidemia in high-risk populations.

### Managing adverse effects of statin therapy in older adults: balancing efficacy and safety

In the analyzed studies, target values were attained through either combination therapy or higher dosages. When comparing outcomes and adverse effects, combination therapy demonstrated a lower incidence of common and undesirable side effects linked to statin usage. Research on statin monotherapy and dose titration indicates that adverse effects can be managed even at elevated doses when patients are not started on the maximum dose immediately but rather titrated to reach the desired outcomes [5, 8, 9, 11].

**Fig. 2:**
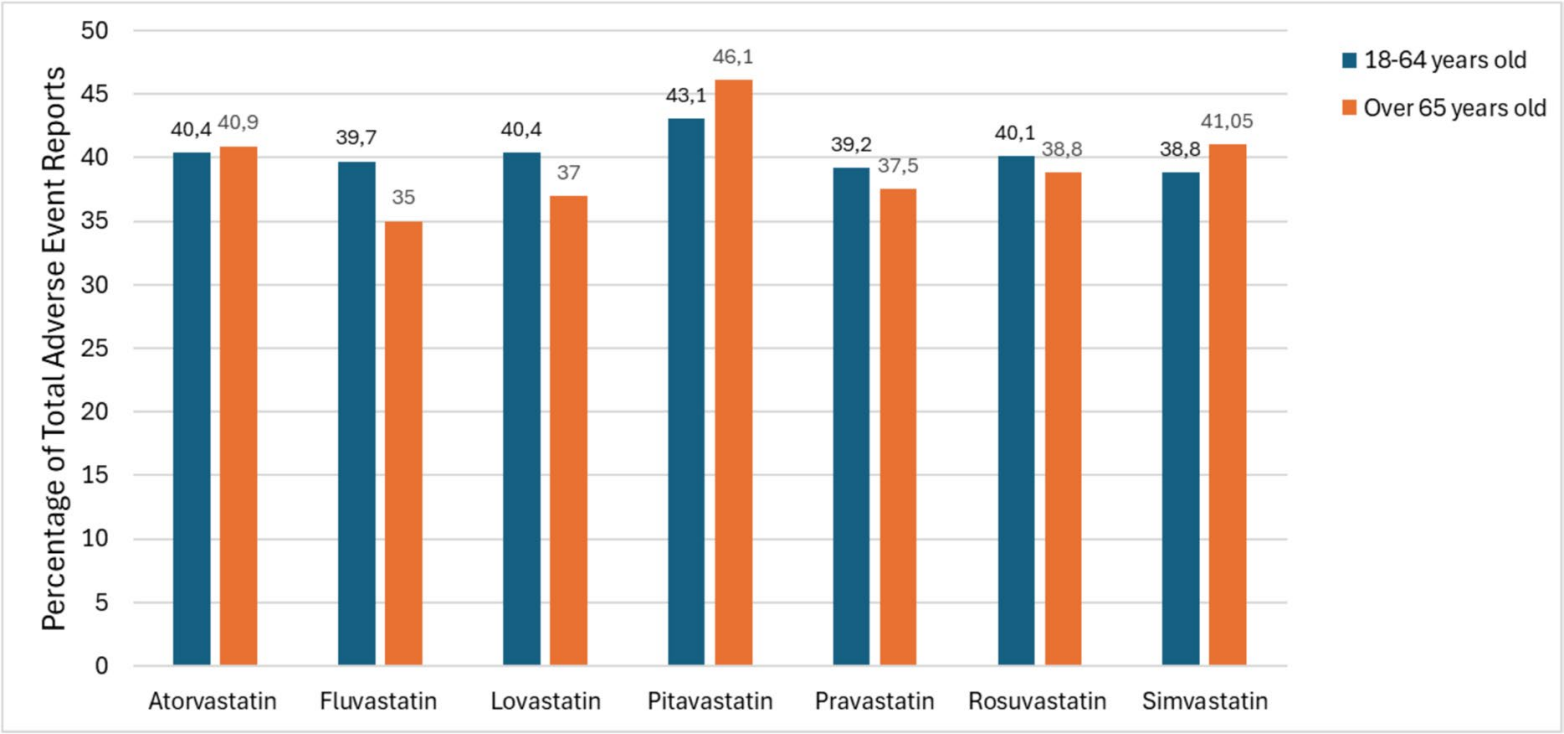
Ratio of the reported musculoskeletal and connective tissue disorders’ adverse reactions in the 18–64 years age group and over 65 years age group and the total number of reported adverse reactions in the last 12 months. *X*-axis: name of the active substances; *Y*-axis: percentage of total adverse event reports. This percentage shows how often a specific adverse event was reported relative to all reported adverse events for a particular drug in each age group [24]

**Fig. 3:**
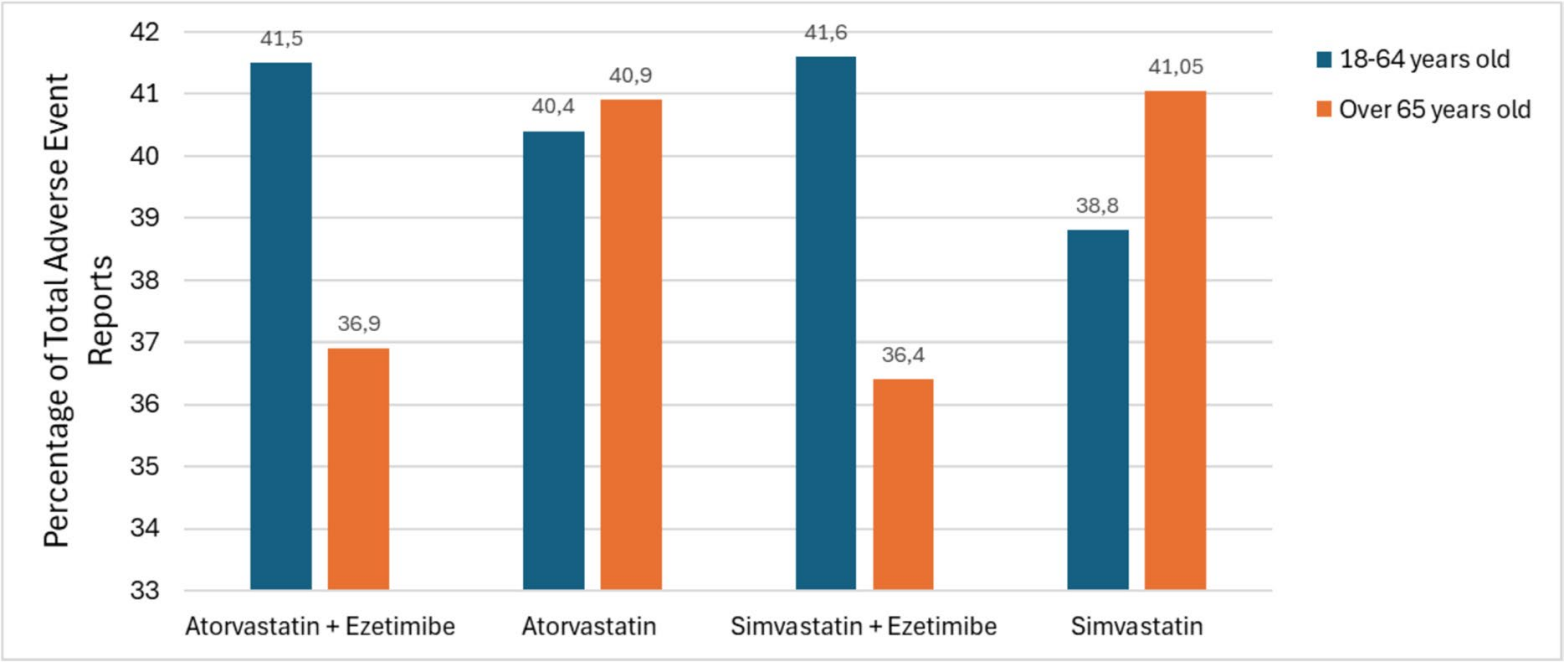
The reported ratio of musculoskeletal and connective tissue disorders’ adverse reactions to atorvastatin, simvastatin, and their combination with ezetimibe in the 18–64 years age group and over 65 years age group, along with the total number of reported adverse reactions in the last 12 months. *X*-axis: name of the active substances; *Y*-axis: percentage of total adverse event reports. This percentage shows how often a specific adverse event was reported relative to all reported adverse events for a particular drug in each age group [24]

Discussing potential side effects, particularly in the population of older adults, is crucial for making informed decisions about medication use. According to data from the EMA EudraVigilance database, musculoskeletal and connective tissue disorders are the most frequently reported adverse reactions linked to statin use. Figure 2 shows the ratio of reported musculoskeletal and connective tissue disorders, adverse reactions per statin for the last 12 months, downloaded from the EMA’s EudraVigilance site [24]. This trend remains consistent even when statins are combined with ezetimibe although the incidence of these reactions is notably lower compared to when statins are used as monotherapy. Figure 3 shows the number of individual cases of reported adverse reactions in the case of atorvastatin and simvastatin and the combination of atorvastatin and ezetimibe and simvastatin and ezetimibe for the last 12 months, downloaded from the EMA’s EudraVigilance site [24]. Combination therapy caused no more musculoskeletal side effects in the over 65 years old age group than shown in Fig. 2.

Myalgia, muscle weakness, and cramps are also common side effects observed in studies related to statin use. For older adults, muscle-related effects are of particular concern, ranging from muscle pain without elevated serum creatinine kinase levels to rhabdomyolysis. However, life-threatening toxicity due to these side effects is a rare occurrence [25]. Therefore, it is essential to weigh the benefits and risks of statin use in the population of older adults, taking into account the potential for muscle-related side effects.

### Patient-centered outcomes: subgroup analysis by age categories and hard clinical outcomes

In the 65–75 age group, Neil et al. focused on patients with type 2 diabetes and found that atorvastatin 10 mg reduced LDL cholesterol levels by 41% compared to placebo, leading to a 38% reduction in initial major cardiovascular events [6]. Constance et al. observed that high-risk patients aged ≥ 65 years achieved superior lipid target reductions with atorvastatin plus ezetimibe compared to atorvastatin titration [11]. Similarly, Stender et al. evaluated the efficacy and safety of pitavastatin in older adults (≥ 65 years), reporting a significant 43% reduction in LDL cholesterol levels after titration [10].

For the 75–85 age group, the ALLIANCE Study included patients aged 65–75 years and demonstrated that high-dose atorvastatin (up to 80 mg) resulted in a 52% reduction in cardiac death and nonfatal myocardial infarction [7]. Additionally, Mack et al. reported that only 31.1% of patients over 75 years in long-term care received statins, with just 5.4% prescribed high-intensity doses. Severe cognitive or functional impairments reduced the likelihood of statin prescriptions [14].

Unfortunately, none of the studies in this review specifically analyzed data for patients aged over 85 years. This reflects a critical gap in the literature and underscores the need for future research to address this underrepresented subgroup.

Although much of the existing research on statin therapy in the populations of older adults emphasizes surrogate endpoints such as lipid level reductions, several studies included in this review reported important hard clinical outcomes, including cardiovascular events and mortality. Neil et al. examined a cohort of patients aged 65 to 75 years with type 2 diabetes and found that atorvastatin 10 mg was associated with a 38% reduction in major cardiovascular events, including myocardial infarction and stroke, compared to placebo [6]. Similarly, the ALLIANCE study demonstrated that high-dose atorvastatin, up to 80 mg, led to a 52% reduction in cardiac death and nonfatal myocardial infarction in patients aged 65 to 75 years [7].

Adherence to statin therapy also played a significant role in clinical outcomes. Musich et al. highlighted that non-adherence in older adults was associated with significantly higher rates of cardiovascular hospitalizations. Even minimal adherence to statin therapy was linked to a lower risk of adverse cardiovascular events compared to non-adherence, underscoring the importance of supporting compliance in this population [13]. Mack et al., although primarily focused on prescribing patterns, found that residents in long-term care settings who received statin therapy had fewer hospitalizations related to cardiovascular events compared to those not on statins [14].

## Discussion

The findings of this review provide critical insights into the efficacy and safety of statin therapy in older adults, emphasizing several key areas. Statin therapy has consistently demonstrated effectiveness in lowering LDL cholesterol and reducing the risk of major cardiovascular events across diverse population of older adults. These effects are especially pronounced when statin therapy is combined with ezetimibe, a strategy that not only enhances lipid-lowering outcomes but also mitigates some common side effects associated with higher statin dosages.

A notable contribution of this review is its focus on dose titration as a strategy to optimize statin therapy in older adults. The studies analyzed demonstrate that aggressive dose titration can achieve target LDL-C levels with minimal adverse events, highlighting the importance of individualized dosing strategies in this population. However, in some studies, because of the short follow-up period, the clinical outcomes were missing and could not support the fact that the therapy could effectively lower the incidents of all cardiovascular outcomes [5, 8–11, 15].

Additionally, the review underscores the critical role of adherence in maximizing the benefits of statin therapy. Even minimal statin use is preferable to no treatment, particularly in high-risk individuals, underscoring the importance of adherence in preventing cardiovascular events.

The review provides a comprehensive analysis of combination therapy, demonstrating that the co-administration of statins and ezetimibe exhibits superior efficacy in achieving lipid targets compared to statin monotherapy, particularly in geriatric populations and individuals at elevated risk for cardiovascular events. This therapeutic combination not only enhances lipid profiles but also maintains a favorable safety profile, rendering it a valuable approach for managing dyslipidemia. The relationship between dietary cholesterol intake and lipid-lowering therapy efficacy is crucial for understanding combination therapy superiority [26–28]. Intestinal cholesterol derives from two primary sources: dietary intake (25%) and biliary cholesterol via enterohepatic circulation (75%) [29]. Ezetimibe targets both pathways by selectively inhibiting the NPC1L1 transporter, which facilitates cholesterol uptake into enterocytes [30]. Clinical evidence demonstrates that ezetimibe reduces fractional cholesterol absorption from approximately 50% to 23%, representing a 54% reduction in absorption efficiency [31]. This mechanism is particularly beneficial for patients with high dietary cholesterol intake, as traditional statins primarily inhibit endogenous cholesterol synthesis without addressing absorption pathways [27, 28]. Importantly, the compensatory increase in cholesterol synthesis observed with ezetimibe monotherapy (approximately 89% increase) is effectively counteracted when combined with statin therapy, eliminating this potential limitation [28].

However, in the very elderly population, the clinical significance of improving lipid profiles warrants careful consideration, given their unique health circumstances, including polypharmacy risks, limited life expectancy, and competing morbidities. A systematic review and meta-analysis of observational studies has demonstrated a statistically significant association between statin therapy and reduced risk of all-cause mortality, cardiovascular death, and stroke in individuals aged 65 and older without pre-existing cardiovascular disease (CVD).

Nevertheless, the same review underscores that the potential benefits of lipid-lowering therapy in very elderly patients may be attenuated due to the aforementioned factors, emphasizing the necessity for personalized treatment strategies in this demographic. This nuanced approach acknowledges the heterogeneity within the population of older adults and the need for evidence-based, patient-centered decision-making in lipid management for older adults [32]. The review further summarized improvements in lipid profiles among the population of older adults included in each study, suggesting that while lipid reduction is achievable, the clinical significance may vary depending on the patient’s overall health status.

The quality of evidence for lipid outcomes in the reviewed studies ranges from moderate to high. The studies consistently demonstrate statistically significant reductions in LDL cholesterol levels among older adults treated with statin therapy, particularly when combined with ezetimibe or dose titration. The evidence is strengthened by precise measurements and direct assessments of lipid profiles. However, the relatively short follow-up periods in some studies slightly reduce the overall quality rating.

For clinical outcomes, the quality of evidence is moderate. The data on long-term clinical endpoints such as cardiovascular events and all-cause mortality are less consistent and direct compared to the lipid outcomes. Several studies lack reporting on these critical long-term outcomes, which limit the ability to draw robust conclusions regarding the sustained clinical benefits of statin therapy in the population of older adults. This gap in evidence constrains the strength of recommendations that can be made concerning the long-term efficacy of statins for reducing cardiovascular morbidity and mortality in older adults.

Adherence to statin therapy plays a crucial role in preventing cardiovascular disease (CVD) events. Studies have shown that patients with low adherence to statins may experience no reduction or even an increase in cardiovascular mortality [33]. High-intensity statins, prescribed for individuals at high risk of CVD, lose their protective effects against cardiovascular events if not taken as prescribed, leading to higher hospitalization rates among nonadherent individuals [34]. Nonadherent individuals face a heightened risk of adverse cardiovascular outcomes, emphasizing the importance of medication adherence in managing and preventing CVD complications [35]. This underscores the critical role of statin adherence in reducing the burden of cardiovascular disease and related hospitalizations. To improve statin adherence in this population, a multifaceted approach is the most effective. Patient education should be clear, accessible, and preferably delivered face-to-face in pharmacy or clinical settings. Even a single session of personalized education, whether provided by a pharmacist or a general practitioner, can lead to meaningful improvements in adherence [36–38]. However, simple reminders alone are not sufficient [39]. Sustained, regular follow-up and delivery in multiple settings or even at home can further enhance effectiveness, although these latter strategies may be more difficult to implement [36]. Fixed-dose combinations also represent a promising approach, as reducing the pill burden in older adults can improve therapeutic outcomes and increase patient safety [40, 41]. However, the long-term outcomes of this approach still need to be investigated [41]. Guidelines from major organizations like the ESC/EAS and AHA/ACC emphasize regular lipid monitoring and adherence to lifestyle changes as vital components of effective statin therapy [3, 4].

Beyond their lipid-lowering effects, statins also exhibit pleiotropic effects, such as improving endothelial function, reducing inflammation, and enhancing vascular function [42]. These additional benefits, as highlighted by recent research, may further enhance the cardioprotective properties of statins by improving plaque stability and reducing oxidative stress, potentially leading to better clinical outcomes for those at risk of cardiovascular disease.

In the context of primary prevention, statins have shown consistent benefits across diverse demographic and clinical characteristics. However, data for individuals aged 75 and above remain limited, and more information is needed on the use of statins in this age group. A recent review highlights the growing importance of such data and new guidelines for older patients [43]. The US Preventive Services Task Force (USPSTF) recommends broader use of statins for primary prevention, particularly in individuals with a 10-year cardiovascular disease risk score below 10% [44]. Ongoing trials, such as the Statin Therapy for Reducing Events in the Elderly (STAREE, NCT02099123), are addressing this gap. The STAREE trial is randomizing 18,000 adults aged 70 or older in Australia without known ASCVD to receive either 40 mg/day of atorvastatin or placebo. The co-primary outcomes are time to death, dementia, significant disability, or a major cardiovascular event (fatal or nonfatal). Similarly, the Pragmatic Evaluation of Events and Benefits of Lipid-Lowering in Older Adults (PREVENTABLE, NCT04262206) is randomizing 20,000 US adults aged 75 and older, living independently and without preexisting cardiovascular disease, to either 40 mg of atorvastatin daily or placebo. PREVENTABLE aims to determine if statin therapy for primary prevention can reduce a composite outcome of death, dementia, and persistent disability, with secondary endpoints including mild cognitive impairment and cardiovascular events [43]. These guidelines stress the importance of shared decision-making, encouraging healthcare providers to discuss the potential benefits and risks of statin therapy with their patients. Future research on this vulnerable population could benefit from observational studies pragmatic and adaptive clinical trials, and community-based participatory research designs [45, 46]. Researchers should carefully minimize the burden on participants, ensure informed consent, provide comprehensive support, and tailor interventions to accommodate varying cognitive and physical abilities. 

Although data from younger elderly populations (such as those aged 65–84 years) can help inform clinical decisions, healthcare providers should be cautious when applying these findings broadly and remain vigilant for potential adverse effects or variations in patient responses. Treatment plans should be individualized based on thorough geriatric assessments that consider functional abilities, existing comorbidities, and patient preferences, especially given the limited robust clinical trial evidence for this age group [47]. Engage in shared decision-making with patients and caregivers, emphasizing quality of life and functional outcomes over disease-oriented targets alone.

Overall, by considering the pleiotropic effects of statins and engaging in informed discussions with patients, healthcare providers can optimize the use of statins in primary prevention strategies, ultimately contributing to the reduction of cardiovascular disease burden and promoting cardiovascular health and well-being.

## Limitations

### Core methodological constraints

This systematic review identified significant methodological limitations affecting the clinical applicability of findings on statin therapy in elderly populations. Substantial heterogeneity in study designs, populations, interventions, and outcome measures precluded meta-analytical synthesis and limited cross-study comparability. Many studies prioritized surrogate lipid markers over clinically relevant cardiovascular events and mortality endpoints—a critical limitation when assessing therapeutic benefit in elderly patients.

The most vulnerable elderly subgroups—those ≥ 85 years, institutionalized individuals, and patients with cognitive impairment—were systematically underrepresented. This exclusion compromises external validity for these high-risk populations who increasingly require cardiovascular interventions. Limited sample sizes in elderly cohorts, particularly beyond age 80, resulted in insufficient statistical power to detect clinically meaningful differences, reducing confidence in therapeutic recommendations.

### Safety and demographic considerations

Safety assessment was hampered by inconsistent adverse event reporting methodologies and predominantly short-term follow-up durations, potentially underestimating delayed complications particularly relevant in geriatric polypharmacy contexts (muscle toxicity, cognitive effects, drug interactions). The absence of standardized adverse event classification and variable monitoring periods complicated risk–benefit synthesis.

Sex-specific analyses were notably scarce despite established differences in statin metabolism, therapeutic response, and adverse effect profiles between women and men—concerning given documented disparities in statin prescription and adherence patterns that may differentially impact outcomes in elderly populations.

### Therapeutic evidence gaps

Evidence for combination lipid-lowering therapy was largely restricted to ezetimibe-based regimens, with insufficient representation of newer agents like PCSK9 inhibitors, despite their potential advantages in elderly patients (reduced drug interactions, alternative mechanisms for statin-intolerant patients).

### Clinical implications

Given the inherent complexity of geriatric populations (variable comorbidities, extensive polypharmacy, heterogeneous frailty), these limitations underscore the need for personalized statin prescribing approaches based on individual characteristics and preferences. Recommendations must be interpreted cautiously, recognizing the need for future research with standardized methodologies, comprehensive outcome reporting, adequate representation of vulnerable elderly subgroups, and sufficient follow-up to inform evidence-based geriatric cardiovascular medicine.

## Conclusion

This systematic review highlights the importance of individualized statin dosing strategies for older adults. Statins remain an essential therapy for reducing cardiovascular disease risk; however, the complexity of aging, comorbidities, and polypharmacy demands a personalized approach to treatment. Evidence from included studies supports dose titration and combination therapies as effective means of achieving lipid targets while mitigating side effects. Adherence strategies are also crucial in optimizing therapeutic outcomes in this population. While these findings underscore the clinical utility of such approaches, they should be interpreted with caution, as highlighted in the limitations—many studies lacked sufficient follow-up, which may affect the strength of the conclusions. Although the review confirms the clinical utility of statins in older adults, there are gaps in evidence regarding patients aged over 85 years and long-term clinical outcomes. Addressing these gaps through future research with robust study designs and extended follow-up periods is essential to inform clinical decision-making further. Clinicians should assess the risks and benefits of statin therapy individually, especially in frail or severely ill patients, engaging in shared decision-making to align treatment plans with patient preferences and health goals.

## Data Availability

All data supporting the findings of this study are fully incorporated within the manuscript.
